# Effects of Phytohormone-Producing Rhizobacteria on Casparian Band Formation, Ion Homeostasis and Salt Tolerance of Durum Wheat

**DOI:** 10.3390/biom12020230

**Published:** 2022-01-29

**Authors:** Elena Martynenko, Tatiana Arkhipova, Vera Safronova, Oksana Seldimirova, Ilshat Galin, Zarina Akhtyamova, Dmitry Veselov, Ruslan Ivanov, Guzel Kudoyarova

**Affiliations:** 1 Ufa Federal Research Centre, Ufa Institute of Biology, RAS, Prospekt Oktyabrya, 69, 450054 Ufa, Russia; evmart08@mail.ru (E.M.); TNArkhipova@mail.ru (T.A.); o_seldimirova@mail.ru (O.S.); ilshat.rafkatovitch@gmail.com (I.G.); akhtyamovazarina@gmail.com (Z.A.); veselov@anrb.ru (D.V.); ivanovirs@mail.ru (R.I.); 2 Russia Research Institute for Agricultural Microbiology, Group of Culture of Beneficial Microorganisms, Podbelskogo sh. 3, Pushkin, 196608 Saint-Petersburg, Russia; v.safronova@rambler.ru

**Keywords:** durum spring wheat, *Triticum durum* Desf., salinity, sodium, potassium, phosphorus, plant growth-promoting rhizobacteria (PGPR), cytokinins, indoleacetic acid

## Abstract

Inoculation with plant growth-promoting rhizobacteria can increase plant salt resistance. We aimed to reveal bacterial effects on the formation of apoplastic barriers and hormone concentration in relation to maintaining ion homeostasis and growth of salt-stressed plants. The rhizosphere of a durum wheat variety was inoculated with cytokinin-producing *Bacillus subtilis* and auxin-producing *Pseudomonas mandelii* strains. Plant growth, deposition of lignin and suberin and concentrations of sodium, potassium, phosphorus and hormones were studied in the plants exposed to salinity. Accumulation of sodium inhibited plant growth accompanied by a decline in potassium in roots and phosphorus in shoots of the salt-stressed plants. Inoculation with both bacterial strains resulted in faster appearance of Casparian bands in root endodermis and an increased growth of salt-stressed plants. *B. subtilis* prevented the decline in both potassium and phosphorus concentrations and increased concentration of cytokinins in salt-stressed plants. *P. mandelii* decreased the level of sodium accumulation and increased the concentration of auxin. Growth promotion was greater in plants inoculated with *B. subtilis.* Increased ion homeostasis may be related to the capacity of bacteria to accelerate the formation of Casparian bands preventing uncontrolled diffusion of solutes through the apoplast. We discuss the relative impacts of the decline in Na accumulation and maintenance of K and P content for growth improvement of salt-stressed plants and their possible relation to the changes in hormone concentration in plants.

## 1. Introduction

Excessive salt concentrations in a soil solution negatively affect plant growth and productivity. These effects arise as a result of water scarcity due to a decrease in the availability of water from saline soil solution and accumulation of sodium ions, being toxic for plants at high concentrations. The areas of saline arable land are growing steadily all over the world, due to increasing climate aridity and expansion of irrigated agriculture [1,2,3]. This determines the search for approaches to increase salt tolerance of plants, i.e., the ability to maintain productivity under salt stress. Recently, the use of plant growth-promoting rhizobacteria (PGPR), which stimulate plant growth and increase their productivity under stress conditions, has become increasingly popular [4,5,6,7,8]. Nevertheless, not all PGPR are capable of increasing salt tolerance. To select more promising strains and optimize biotechnology of their use as regulators of plant salt tolerance, it is important to clearly understand how their effect on plant salt tolerance is realized. The interaction mechanisms between PGPR and plants are being intensively studied [9,10]. Many PGPR have been investigated for their role in improving water relations, ion homeostasis and photosynthetic efficiency in plants under salt stress. Nevertheless, the mechanisms of bacterium-induced increases in salt tolerance of plants are complex and insufficiently studied [6].

Disturbance of ion homeostasis is one of the main reasons for the growth-inhibiting effect of salinity [11,12]. Competition for molecular transporters between sodium and potassium ions reduces influx of the latter [13] and disturbs plant metabolism [11]. A compensatory upregulation of genes encoding potassium transporters is one of the mechanisms that maintain ion homeostasis during salinity [14,15]. Under salinity stress, inoculation with PGPR (*Pseudomonas fluorescens* biotype F, *P. fluorescens* CECT 378T, *Bacillus tequilensis*, *B. aryabhattai*, *Providencia stuartii*, *Pantoea agglomerans* or *Arthrobacter* sp.) resulted in an increased K^+^/Na^+^ ratio [16], attributed to upregulation of genes responsible for ion transport. Comparative transcriptomics revealed upregulation of Na^+^ transporter *HKT1*, as well as the Na^+^/H^+^ antiporters *NHX1* and *NHX2* due to inoculation of a *Pseudomonas oryzihabitans* strain, indicating a putative improved mechanism of preventing the accumulation of excess Na^+^ in photosynthetic tissues [17].

Salinity promotes the formation of secondary walls and Casparian bands [18]. These effects play an important role in restricting the non-selective apoplastic bypass of salts into the stele, thereby forcing solutes to move through the selectively permeable membrane channels [19]. A recent review emphasizes that Casparian bands play a key role in ion exclusion and they should be primary targets for breeding salt-tolerant crops [20]. However, the formation of Casparian bands in relation to bacterial action on plants was considered only as a mechanism preventing the entry of pathogenic bacteria into plants [21]. It was shown that overexpression of class III peroxidase enhanced lignification as an apoplastic barrier for infection [22]. Nevertheless, the possible promotion of apoplast barrier formation by bacteria as a mechanism of a bacterium-induced increase in salt resistance has never been addressed.

It is important to understand how bacteria induce the processes resulting in increased salt tolerance. In plants, hormones play the role of signals that trigger various processes, and PGPR are able to synthesize plant hormones [6,8,23,24]. At the same time, the role of bacterial hormones is discussed mainly in connection with their immediate effect on plant growth, and the possible role of hormones in triggering other processes enabling salt resistance is only assumed [25]. Root-synthesized cytokinins (hormones also produced by bacteria) have been shown to reduce the extent of the decline in the concentration of potassium and to improve shoot growth in salt-stressed tomato plants [26]. These data indicate possible involvement of hormones in triggering bacterium-induced processes that increase ion homeostasis during salinity stress.

The purpose of this work was to study the effects of PGPR producing plant hormones on the formation of secondary walls and Casparian bands in durum wheat plants in relation to the changes in concentration of sodium, potassium, phosphorus and hormones and plant salt resistance. The selected PGPR strains have been previously shown to produce cytokinins [27] and auxin [28] and increase growth and productivity of salt-stressed wheat in field experiments [29]. However, the importance of the formation of Casparian bands and changes in hormonal and ion concentrations for the observed effects of bacterial inoculation on plant salt resistance has not been addressed.

## 2. Materials and Methods

### 2.1. Plant Material, Bacterial Strains and Culture Media

The durum spring wheat *Triticum durum* Desf. variety Bashkirskaya 27 was studied. The Gram-positive aerobic spore-forming cytokinin-producing bacterium *Bacillus subtilis* IB-22 (GenBank MT590663) [27] and Gram-negative auxin-producing bacterium *Pseudomonas mandelii* IB-Ki14 (All-Russian Collection of Microorganisms B-3250) [28] from the collection of microorganisms of the Ufa Institute of Biology of the UFIC RAS (Ufa, Russia) were used for inoculation of plants. Both bacterial strains are moderate halophiles and tolerant to 5–7% NaCl in vitro. Bacterial inoculum was prepared by cultivation of *B. subtilis* IB-22 on K1G medium containing 1% starch, 0.3% peptone, 0.3% yeast extract, 0.3% maize extract, 0.2% K_2_HPO_4_ and 0.2% (NH_4_)_2_SO_4_ and *P. mandelii* IB-Ki14 on King’s B medium (2% peptone, 1% glycerol, 0.15% K_2_HPO_4_, 0.15% MgSO_4_ × 7H_2_O) [29], respectively. Bacteria were cultured in Erlenmeyer flasks for 72 h at 37 °C or for 48 h at 28 °C for *B. subtilis* IB-22 or *P. mandelii* IB-Ki14, respectively.

### 2.2. Experimental Design

To ensure drainage, a layer of gravel was placed at the bottom of vessels with a volume of 500 cm^3^. After installing a glass tube for gas exchange, the pots (500 mL) were filled with 0.45 kg of dry soil from humus-rich clay-illuvial horizon with medium humus content (6.3%) and supplemented with 10% sand. Three days before the experiments, the soil in the pots was supplemented with water or with a 100 mM NaCl solution up to 100% of the whole field capacity. Wheat seeds were sterilized by soaking in a solution of 96% ethanol/3% H_2_O_2_ (1:1, *v*/*v*) for 5 min and then repeatedly washed with distilled water. Ten wheat seeds were placed in each vessel and inoculated with 1 mL of the bacterial suspension per seed (10^7^ CFU/mL). Plants were grown at 24 °C, 420 µmol m^−2^ s^−1^ PAR irradiance and 14 h light/10 h dark photoperiod. Plants grown in soil without the introduced bacteria were used as a control. The soil moisture was maintained at 70% of the whole field capacity by watering the vessels daily with distilled water. The amount of water required for irrigation was calculated by weighing the vessels.

### 2.3. Elemental Analysis

Concentrations of sodium, potassium and phosphorus in the roots and the 1st and 2nd mature leaves of wheat plants were assessed as previously described [30] using an ICPE-9000 inductively coupled plasma emission spectrometer (Shimadzu, Kyoto, Japan). For this purpose, plant samples were digested in a mixture of concentrated HNO_3_ and 38% H_2_O_2_ at 70 °C using a DigiBlock digester (LabTech, Sorisole, Italy).

### 2.4. Determination of Hormones

To determine the content of hormones, a sample of 5 plants was taken for each biological replicate (roots or shoots) on the sixth and eleventh days after the start of experiments. Hormones were extracted with 80% ethanol (1:10) overnight. Extract separated by filtration was evaporated to an aqueous residue and divided into two parts for determination of cytokinins and indoleacetic acid (IAA, a hormone from the class of auxins). Cytokinins from the aqueous residue after their concentration on a C-18 cartridge (Waters Corporation, Milford, MA, USA) were separated by thin layer chromatography as previously described [31]. Contents of zeatin, its riboside and nucleotide in the corresponding chromatographic zones were determined by enzyme-linked immunosorbent assay (ELISA) using antibodies against zeatin riboside [32]. IAA was partitioned from the acidified aqueous residue with diethyl ether as previously described [28]. After methylation of samples, the IAA concentration in the extract was determined by ELISA using the appropriate specific antibodies [33].

### 2.5. Measurement of Photosynthetic Activity

The chlorophyll fluorescence of intact leaves was measured with the Junior PAM fluorometer (Walz, Effeltrich, Germany) using the WinControl 3 software. Before measurements, the plants were kept in the dark for 30 min. The maximum photochemical quantum yield of photosystem 2 was calculated using the formula Fv/Fm = (Fm − Fo)/Fm, where Fm is the maximum fluorescence yield of chlorophyll in dark-adapted leaves in response to a flash of saturating light, and Fo is the minimum fluorescence yield of chlorophyll at the turning on low-intensity modulated light in dark-adapted leaves.

### 2.6. Visualization of Lignin and Suberin

To visualize lignin and suberin with berberine hemisulfate [34], cross sections were cut by hand from the segments of the basal part of the roots with a safety razor on the sixth and eleventh days after the start of experiments. Sections were stained using an aqueous solution of berberine hemisulfate (0.1% *w*/*v*) for 1 h, rinsed 2 times with distilled water, then, to enhance the fluorescence intensity, sections were additionally stained for 15 min with toluidine blue (0.05% *w*/*v*) in 0.1 M phosphate buffer (pH 5.6), rinsed 2 times with distilled water, embedded in a 0.1% FeCl_3_/50% glycerol mixture and covered with a cover slip. Fluorescence of berberine was excited with a 488 nm solid-state laser using an Olympus FluoView FV3000 confocal laser scanning confocal microscope (Olympus, Tokyo, Japan).

### 2.7. Statistics

The data were statistically processed using standard MS Excel programs. The tables and figures show means and their standard errors (SEs). The significance of differences was assessed by ANOVA followed by Duncan’s test (*p* ≤ 0.05).

## 3. Results

### 3.1. Lignifications of Xylem Cell Walls and Formation of Casparian Bands

**Figure 1:**
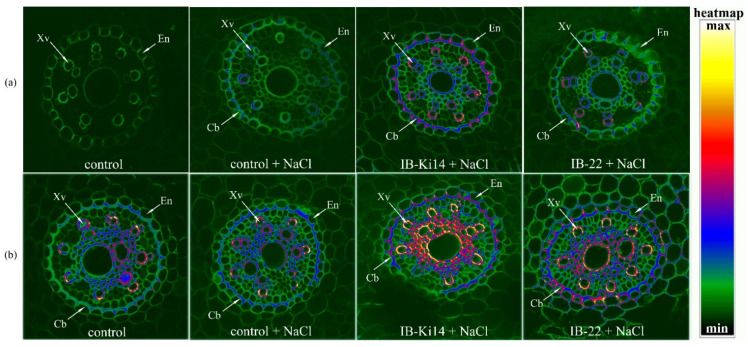
Localization of lignin and suberin and detection of Casparian bands in berberine-stained root cross-sections of the 6-day-old (**a**) and 11-day-old (**b**) wheat plants treated with NaCl and bacterial strains (*Pseudomonas mandelii* IB-Ki14 (IB-Ki14) and *Bacillus subtilis* IB-22 (IB-22)). Cb—Casparian bands, En—endoderm, Xv—xylem vessels. The heatmap shows color-coded fluorescence signal intensities.

On the 6th day after bacterial and salt treatment in the absence of NaCl, a low level of fluorescence was registered in either the xylem vessels or endodermis cells of berberine-stained roots of plants untreated with bacteria, suggesting a low level of secondary wall formation (Figure 1a).

Salinity increased fluorescence of xylem cell walls and made endodermis cell walls thicker compared to control plants (without bacterial or NaCl treatment). Fluorescence of the cells walls of xylem vessels was increased by treatment with bacteria, indicated by their red coloring, while endodermis cell walls became thicker, indicating accelerated formation of secondary walls by this bacterial strain, and fluorescence was detected in the lamella of endodermis, marking the formation of Casparian bands. The effect was most noticeable in the case of plants treated with *Pseudomonas mandelii* IB-Ki14.

Later, on the 11th day after bacterial and salt treatment, increased staining of xylem cells and endodermis of control plants suggested lignification of xylem cell walls and formation of Casparian bands (Figure 1b). Heat map coloring showed that salinity increased the intensity of the fluorescent signal in the endodermis. Bacterial treatment increased fluorescence intensity most noticeably in the lamella.

### 3.2. Effects of Salinity and Bacteria on the Mass of Plants

Salinity decreased shoot and root mass by approximately 35 and 25%, respectively, compared to the unstressed control (Table 1). Under salinity, inoculation of plants with *P. mandelii* IB-Ki14 and *B. subtilis* IB-22 strains increased the shoot mass by 25 and 30% and root mass by 10 and 15%, respectively.

The shoot mass increment was greater in the case of *B. subtilis* IB-22 than the *P. mandelii* IB-Ki14 strain. A significant increase in root mass was detected only in the case of *B. subtilis* IB-22; plants treated with *P. mandelii* IB-Ki14 occupied an intermediate position between plants untreated with bacteria and those treated with *B. subtilis* IB-22. Thus, bacilli exhibited a greater ability to increase the salt tolerance of durum wheat plants in terms of maintaining plant growth.

### 3.3. Effects of Bacteria on the Hormone Concentration in the Plants

**Figure 2:**
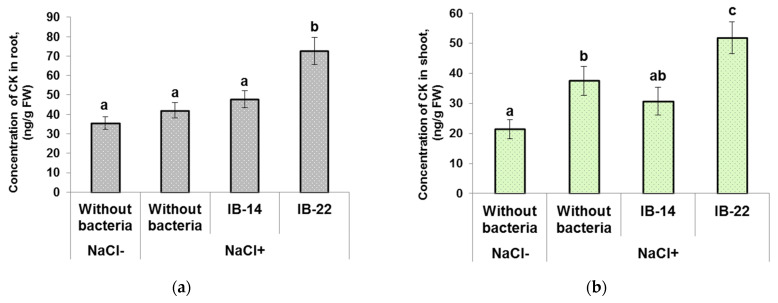
Effect of 100mM NaCl and bacterial treatment with *Pseudomonas mandelii* IB-Ki14 (IB-14) and *Bacillus subtilis* IB-22 (IB-22) on cytokinin concentration in the roots (**a**) and in the shoots (**b**) of 6-day-old wheat plants. Data are means ± SE. Significantly different means are labeled with different letters at *p* ≤ 0.05, *n* = 6 (ANOVA followed by Duncan’s test).

Total content of analyzed cytokinins (zeatin, its riboside and nucleotide) was significantly higher in the roots of plants inoculated with *B. subtilis* IB-22 (Figure 2a). This strain was previously shown to produce cytokinins [27].

Concentration of cytokinins in the shoots increased under the influence of salinity. In shoots of salt-stressed plants, a higher level of cytokinins was detected in plants treated with *B. subtilis* IB-22 compared to non-inoculated plants and those inoculated with *P. mandelii* IB-Ki14 (Figure 2b). Thus, the increase in the level of cytokinins in inoculated plants (both in shoots and roots) was noticeable in the case of cytokinin-producing *B. subtilis* IB-22 and not in auxin-producing *P. mandelii* IB-Ki14.

**Figure 3:**
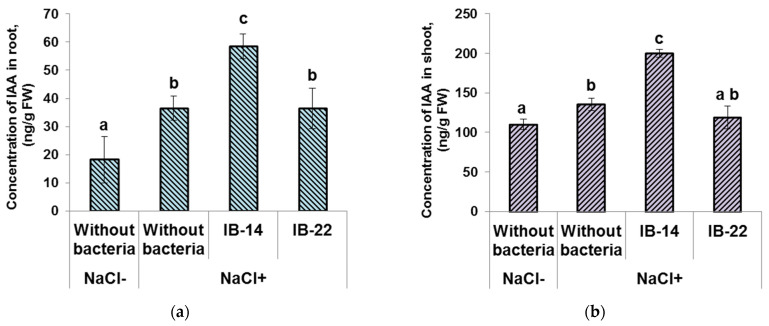
Effect of 100mM NaCl and bacterial treatment with *Pseudomonas mandelii* IB-Ki14 (IB-14) and *Bacillus subtilis* IB-22 (IB-22) on indole-3-acetic acid concentration in the roots (**a**) and in the shoots (**b**) of 6-day-old wheat plants. Data are means ± SE. Significantly different means are labeled with different letters at *p* ≤ 0.05, *n* = 6 (ANOVA followed by Duncan’s test).

Root IAA concentration was increased by salinity. This parameter was higher in plants inoculated with *P. mandelii* IB-Ki14 (Figure 3a) compared to both unstressed and salt-stressed non-inoculated plants, while the concentration of this hormone was similar in non-inoculated plants and those inoculated with *B. subtilis* IB-22 under salinity. The *P. mandelii* IB-Ki14 strain was previously shown to produce auxins [28].

Increased shoot IAA concentration was significant in plants treated with *P. mandelii* IB-Ki14. There was no increase in the level of auxin in the shoots of plants inoculated with cytokinin-producing *B. subtilis* IB-22 (Figure 3b).

Increased concentrations of IAA in plants inoculated with *P. mandelii* IB-Ki14 and cytokinins in those treated with *B. subtilis* IB-22 were also registered later (data not shown).

### 3.4. Elemental Analysis

The presence of NaCl in the soil led to a more than 3-fold increase in sodium concentration both in shoots and roots compared to the control (i.e., plants grown in the soil without NaCl), which was expected (Table 2).

Sodium concentration was about two times higher in the shoots than in the roots of the salt-stressed plants. Inoculation with *P. mandelii* IB-Ki14 decreased sodium accumulation in the shoot. The tendency of a bacterium-induced decrease in root Na^+^ accumulation was statistically insignificant.

At the same time, salinity decreased potassium concentration in the roots by about 25% in both plants not inoculated with bacteria and in those treated with *P. mandelii* IB-Ki14 (Table 2), while in the roots of plants inoculated with *B. subtilis* IB-22, the concentration of the essential element was at the level of the control (non-inoculated plants grown without NaCl). No significant changes in potassium concentration caused by inoculation were detected in shoots.

Salinity led to a significant decrease in the level of phosphorus in the shoots, and only in plants inoculated with the *B. subtilis* IB-22 was this maintained at the control level (non-inoculated plants grown in the absence of salinity) (Table 2).

Neither salinity nor inoculation had a significant effect on the Fv/Fm ratio and it was about 0.790 ± 0.005 on average.

## 4. Discussion

### 4.1. Effect of Salt Stress and Bacterial Inoculation on Sodium Concentration in Wheat Plants and Its Relation to Formation of Apoplast Barriers

As expected, the presence of NaCl in the soil solution increased sodium concentration in durum wheat (Table 2). Na^+^ concentration was higher in the shoots than in roots of salt-treated plants, which is explainable by the low ability of plants of this species to prevent the influx of sodium ions into the shoot [35]. Nevertheless, sodium accumulation was lowered by plant inoculation with *Pseudomonas mandelii* IB-Ki14, which is in accordance with reports on the ability of PGPR to reduce accumulation of these toxic ions in plants [36,37].

Reported cases in halophytes and in some crop plants cited in a recent review [20] showed that apoplastic barriers, including Casparian bands, play an important role in Na^+^ and Cl^−^ exclusion. The present experiments confirm that salinity accelerates the formation of secondary walls of xylem vessels and deposition of suberin and lignin in Casparian bands of the root endodermis of durum wheat plants. While almost no berberine staining was detected in 6-day-old control (unstressed) plants, lignin staining of xylem vessels and the appearance of the first signs of Casparian bands were noticeable in the roots of salt-stressed plants (Figure 1a). Fluorescence corresponding to the deposition of suberin and lignin was enhanced by bacterial treatment in accordance with our suggestion about the capacity of bacteria to increase the deposition of lignin and suberin and formation of apoplast barriers limiting uncontrolled solute movement into xylem. Lignin-based barriers in leaves have been shown to restrict pathogens to the infection site and to confer resistance in plants [38]. However, we failed to find reports showing bacterial effects on the formation of Casparian bands in salt-stressed plants and we are the first to demonstrate this effect. Our results were supported by transcriptome profiling in response to rhizobacteria showing bacterial upregulation of a CASP-like protein gene 4D1, which is a member of the Casparian strip membrane domain protein family [39]. The importance of Casparian band formation for sodium exclusion was confirmed by comparing the effects of *Pseudomonas* and *Bacillus* strains. It showed that sodium accumulation was lower in plants treated with *Pseudomonas mandelii* IB-Ki14, where enhanced formation of Casparian bands was most noticeable. It is important that this bacterium produced auxins [28] and increased auxin concentration in the roots (Figure 3a). A recent report has shown the need for auxin-mediated transcriptional responses to modify suberin synthesis [40], suggesting that bacterium-induced auxin accumulation is involved in the accelerated suberin deposition in Casparian bands.

### 4.2. Relative Importance of Bacterial Control of Na, K and P Levels for Promotion of Salt-Stressed Plant Growth and Its Possible Dependence on Bacterial Effects on Plant Hormones

Although treatment with *P. mandelii* IB-Ki14 reduced sodium accumulation in shoots, which could promote the shoot growth of salt-stressed plants (Table 2), the growth promotion by this strain was less than in the case of *B. subtilis* IB-22, which did not influence the accumulation of sodium in shoots. These results are consistent with the reports showing the lack of a significant correlation between shoot Na^+^ exclusion ability and salt resistance in a broad range of plants [41]. In addition, PGPR have a set of beneficial traits affecting plant growth that could also explain the discrepancy between sodium accumulation and shoot growth promotion. 

Accumulation of sodium ions reduces the activity of photosynthesis [6,11]. Nevertheless, we did not find a decrease in the efficiency of photosynthesis in salt-stressed plants which may be a consequence of sequestering Na^+^ in vacuoles, being characteristic of durum wheat plants [42]. Yet, despite the ability of plants to maintain activity of the photosystem under moderate salinity applied in the present experiments, its growth-inhibiting effect was manifested in a significant decrease in the biomass of both shoots and roots (Table 1). Inhibition of plant growth under the influence of salinity could be a consequence of the detected decline in the concentration of potassium in roots (Table 2) and phosphorus in shoots (Table 2), since maintaining optimal concentration of potassium and phosphorus is essential for plant growth [43]. Bacterial inoculation of salt-stressed plants activated their growth accompanied by bacterium-induced changes in phosphorus and potassium concentrations. The increased potassium concentration in roots and phosphorus in shoots was the most noticeable effect in plants treated with *B. subtilis* IB-22. These effects corresponded to the greater ability of *B. subtilis* IB-22 to stimulate growth of salt-stressed durum wheat plants. Our results indicate that maintaining concentrations of potassium and phosphorus, detected in salt-stressed plants inoculated with *B. subtilis* IB-22, may be more important for promotion of plant growth under salinity than a decline in the accumulation of sodium per se.

As indicated in the Introduction section, it was of interest to identify the putative relationship between the effects of bacteria on plant salt resistance and their ability to produce hormones and influence their concentration in plants. The possible importance of auxin production by *P. mandelii* IB-Ki14 has been discussed above. The observed increase in the level of cytokinins in plants inoculated with *B. subtilis* IB-22 is a predictable effect, since this strain was selected as a cytokinin-producing PGPR [27].

Importantly, only *B. subtilis* IB-22, capable of producing cytokinins [27] and increasing cytokinin levels in the plants, prevented a decline in potassium concentration in salt-stressed plants, indicating that an increase in the content of cytokinins in plants under the influence of inoculation with bacteria can play an important role in maintaining ionic homeostasis. This assumption is consistent with the literature data showing that an increase in cytokinin concentration in transgenic tomato plants reduced the extent of the decline in the concentration of potassium in the salt-stressed plants [26].

Using a similar experimental model, we have recently shown that bacterization leads to an increase in the concentration of abscisic acid (ABA) in the roots of salt-stressed plants [29,44]. In those experiments, the highest level of ABA was recorded in plants inoculated with *B. subtilis* IB-22. Accumulation of ABA in plants treated with *B. subtilis* IB-22 was shown to be due to upregulation of the ABA synthesis gene *HvNCED2* and downregulation of the ABA catabolic gene *HvCYP707A1* [44]. The role of this hormone in the regulation of ion homeostasis in salt-stressed plants is being actively discussed [6,45] and deserves further study.

## 5. Conclusions

A recent review showed that Casparian bands play a key role in salt tolerance [20]. As far as we know, we are the first to reveal bacterium-induced acceleration of deposition of suberin and lignin in Casparian bands of root endodermis in salt-stressed plants. The importance of these effects for limiting uncontrolled diffusion of solutes through apoplasta was supported by the data showing decreased sodium accumulation in the shoots of plants treated with *P. mandelii* IB-Ki14 and maintenance of potassium in the roots and phosphorus contents in the shoots of plants treated with *B. subtilis* IB-22. Although deposition of lignin was greater and faster in roots of plants inoculated with *P. mandelii* IB-Ki14, resulting in decreased Na concentration, growth promotion was greater in salt-stressed plants treated with *B. subtilis* IB-22. This comparison indicates that maintaining concentrations of potassium and phosphorus may be more important for promotion of durum wheat growth under salinity than a decline in the accumulation of sodium per se. The revealed increase in the concentration of cytokinins and auxins in plants treated with *B. subtilis* IB-22 and *P. mandelii* IB-Ki14 and, correspondingly, detected in the present experiments, may be explained by the ability of *B. subtilis* IB-22 to produce cytokinins and *P. mandelii* IB-Ki14 to synthesize auxins reported by us previously [27,28]. The effects of PGPR on plant hormonal regulation are important not only in directly promoting plant growth, but also in other aspects of PGPR actions in plants [25]. Based on the literature data showing that auxin-mediated transcriptional responses modified suberin synthesis [40], while an increase in cytokinin concentration reduced the extent of the decline in the concentration of potassium in the salt-stressed plants [26], we hypothesize that the increased concentration of cytokinins and auxins in plants treated with bacteria may be involved in the increase in salt tolerance. Although this assumption needs further confirmation, the relationships found may draw the attention of researchers to studying the role of bacterium-induced changes in hormone concentration to accelerate the formation of Casparian bands and ion homeostasis in salt-stressed plants.

## Figures and Tables

**Table 1:** Fresh weight of root (*n* = 10) and shoot (*n* = 40) of wheat plants on the 12th day of experiments.

NaCl Concentration, mM	Inoculation	Fresh Mass, mg	
		Root	Shoot
0	Without bacteria	94 ± 6.4 ^b^	305 ± 8.3 ^f^
100	Without bacteria	70 ± 3.3 ^a^	198 ± 7.7 ^c^
100	*P. mandelii* IB-Ki14	77 ± 3.8 ^ab^	243 ± 6.9 ^d^
100	*B. subtilis* IB-22	81 ± 4.5 ^b^	266 ± 6.6 ^e^

Data are means ± SE. Significantly different means are marked with different letters at *p* ≤ 0.05 (ANOVA followed by Duncan’s test).

**Table 2:** Effect of 100mM NaCl and bacterial treatment with *Pseudomonas mandelii* IB-Ki14 and *Bacillus subtilis* IB-22 on sodium, potassium and phosphorus concentration in the roots and in the shoots of 11-day-old wheat plants.

	Treatment	Sodium Concentration, mg/g DW	Potassium Concentration, mg/g DW	Phosphorus Concentration, mg/g DW
Roots	Without bacteria, NaCl−	1.24 ± 0.21 ^a^	10.6 ± 0.8 ^b^	33.5 ± 1.1 ^a^
	Without bacteria, NaCl+	4.48 ± 0.42 ^b^	7.8 ± 0.8 ^a^	33.2 ± 2.7 ^a^
	*P. mandelii* IB-Ki14, NaCl+	4.29 ± 0.27 ^b^	7.8 ± 0.5 ^a^	40.3 ± 1.2 ^b^
	*B. subtilis* IB-22, NaCl+	3.99 ± 0.47 ^b^	10.2 ± 0.1 ^b^	36.1 ± 0.6 ^a^
Shoots	Without bacteria, NaCl−	2.81 ± 0.07 ^b^	30.9 ± 0.6 ^cd^	86.3 ± 1.7 ^d^
	Without bacteria, NaCl+	10.13 ± 0.08 ^d^	30.3 ± 0.8 ^cd^	63.0 ± 2.1 ^c^
	*P. mandelii* IB-Ki14, NaCl+	8.11 ± 0.39 ^c^	29.1 ± 0.6 ^c^	60.0 ± 0.4 ^c^
	*B. subtilis* IB-22, NaCl+	9.85 ± 0.57 ^d^	31.7 ± 0.5 ^d^	85.7 ± 1.6 ^d^

Data are means ± SE. Significantly different means are marked with different letters at *p* ≤ 0.05, *n* = 6 in each column (ANOVA followed by Duncan’s test).
